# A one‐day journey to the suburbs: circadian clock in the *Drosophila* visual system

**DOI:** 10.1111/febs.17317

**Published:** 2024-11-01

**Authors:** Milena Damulewicz, Gabriella M. Mazzotta

**Affiliations:** ^1^ Department of Cell Biology and Imaging Jagiellonian University Kraków Poland; ^2^ Department of Biology University of Padua Padua Italy

**Keywords:** biological clocks, circadian rhythms, *Drosophila*, glia, photoreceptors, visual system

## Abstract

Living organisms, which are constantly exposed to cyclical variations in their environment, need a high degree of plasticity in their visual system to respond to daily and seasonal fluctuations in lighting conditions. In *Drosophila melanogaster*, the visual system is a complex tissue comprising different photoreception structures that exhibit daily rhythms in gene expression, cell morphology, and synaptic plasticity, regulated by both the central and peripheral clocks. In this review, we briefly summarize the structure of the circadian clock and the visual system in *Drosophila* and comprehensively describe circadian oscillations in visual structures, from molecules to behaviors, which are fundamental for the fine‐tuning of visual sensitivity. We also compare some features of the rhythmicity in the visual system with that of the central pacemaker and hypothesize about the differences in the regulatory signals and mechanisms that control these two clocks.

## Graphical abstract

In *Drosophila*, daily rhythms have been identified in the fly visual structures, in terms of gene expression, morphology, and behavior. A functional circadian clock in the retina is important not just for the fine‐tuning of visual sensitivity, but also for the proper maintenance of neuronal integrity.

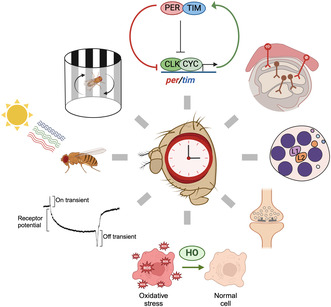

**Abbreviations**
ATP: adenosine triphosphate
Atpα: α subunit of Na+/K + ‐ATPase
BDBT: BRIDE of DOUBLETIME
BRP: Bruchpilot
CDNF: cerebral dopamine neurotrophic factor
CK2: casein kinase 2
CLK: CLOCK
CRY: CRYPTOCHROME
CT: circadian time
CYC: CYCLE
DBT: DOUBLETIME
DD: constant darkness
DN: dorsal neurons
EYA: EYE ABSENT
FAD: flavin adenine dinucleotide
GABA: γ‐aminobutyric acid
H‐B: Hofbauer–Buchner
HO: heme oxygenase
JET: JETLAG
LD: light–dark
LNds: dorsal lateral neurons
LNPs: posterior lateral neurons
LNvs: ventral lateral neurons
MANF: mesencephalic astrocyte‐derived neurotrophic factor
MTHF: methylenetetrahydrofolate
NO: nitric oxide
norpA: no receptor potential A
PER: PERIOD
RdgB: retinal degeneration B
Rh: rhodopsin
SGG: SHAGGY
SnPPIX: Tin protoporphyrin IX dichloride
TF: transcription factor
TIM: TIMELESS
TRP: transient receptor potential
TTFL: transcriptional–translational feedback loop
ZT: Zeitgeber time

## Introduction

The circadian clock is an endogenous timekeeper mechanism that controls biological rhythms for approximately 24 h. It allows organisms to anticipate cycles in their surroundings and synchronize their internal cellular physiology with external environmental stimuli.

At the molecular level, circadian oscillations are generated by an evolutionarily conserved transcriptional–translational feedback system (TTFL): positive elements stimulate the rhythmic transcription of negative elements, which in turn suppresses the activity of positive elements.

The circadian oscillator in *Drosophila melanogaster* is composed of three TTFLs, interlocked by the assembly of the heterodimeric transcription factor CLOCK/CYCLE (CLK/CYC), which provides stability and robustness to the molecular clock [1].

In the ‘core’ *period (per)/timeless (tim)* loop, CLK/CYC heterodimers activate, in the second half of the day, the transcription of *per* and *tim* by binding their promoters at specific sites (CACGTG), called E‐boxes [2, 3] *per* and *tim* mRNA start to accumulate with a peak early in the evening, while the corresponding proteins PER and TIM reach the maximum only in the middle‐late night, approximately 4–6 h after *per* and *tim* mRNA peaks [4, 5, 6, 7]. Soon after translation, PER is phosphorylated and degraded by the combined action of DOUBLETIME (DBT) kinase [8], its mediator BRIDE of DOUBLETIME (BDBT) [9] and SLIMB, a F‐box/WD40‐repeat protein functioning in the ubiquitin–proteasome pathway [10]. PER phosphorylation is counterbalanced by dephosphorylation mediated by protein phosphatase 2A (PP2A) [11]. In addition, TIM levels increase, and the protein binds to the PER/DBT complex, promoting its accumulation and stabilizing it.

PER/TIM/DBT enters the nucleus, promoted by additional kinases acting either on PER, such as casein kinase 2 (CK2), or on TIM, such as SHAGGY (SGG) [12]. By binding to CLK/CYC and removing it from the E‐box, the PER/TIM/DBT complex inhibits *tim* and *per* transcription. Tyrosine phosphorylation of TIM causes its degradation (at least in reaction to light), whereas DBT phosphorylation destabilizes and degrades PER and CLK. [13, 14]. An accumulation of nonphosphorylated (or hypo‐phosphorylated) CLK results in the heterodimerization with CYC and another cycle of *per* and *tim* transcription [6].

The light‐dependent synchronization of this molecular oscillation is mediated by CRYPTOCHROME (CRY) [15], a blue light photoreceptor protein that can bind flavin (FAD) and methyltetrahydrofolate (MTHF) with an absorbance of 420 nm [16]. After light exposure, it binds TIM [17], promoting its degradation through the proteasome with the help of the F‐box protein JETLAG [18]. CRY is also rapidly degraded in the presence of light through the proteasome.

**Fig. 1:**
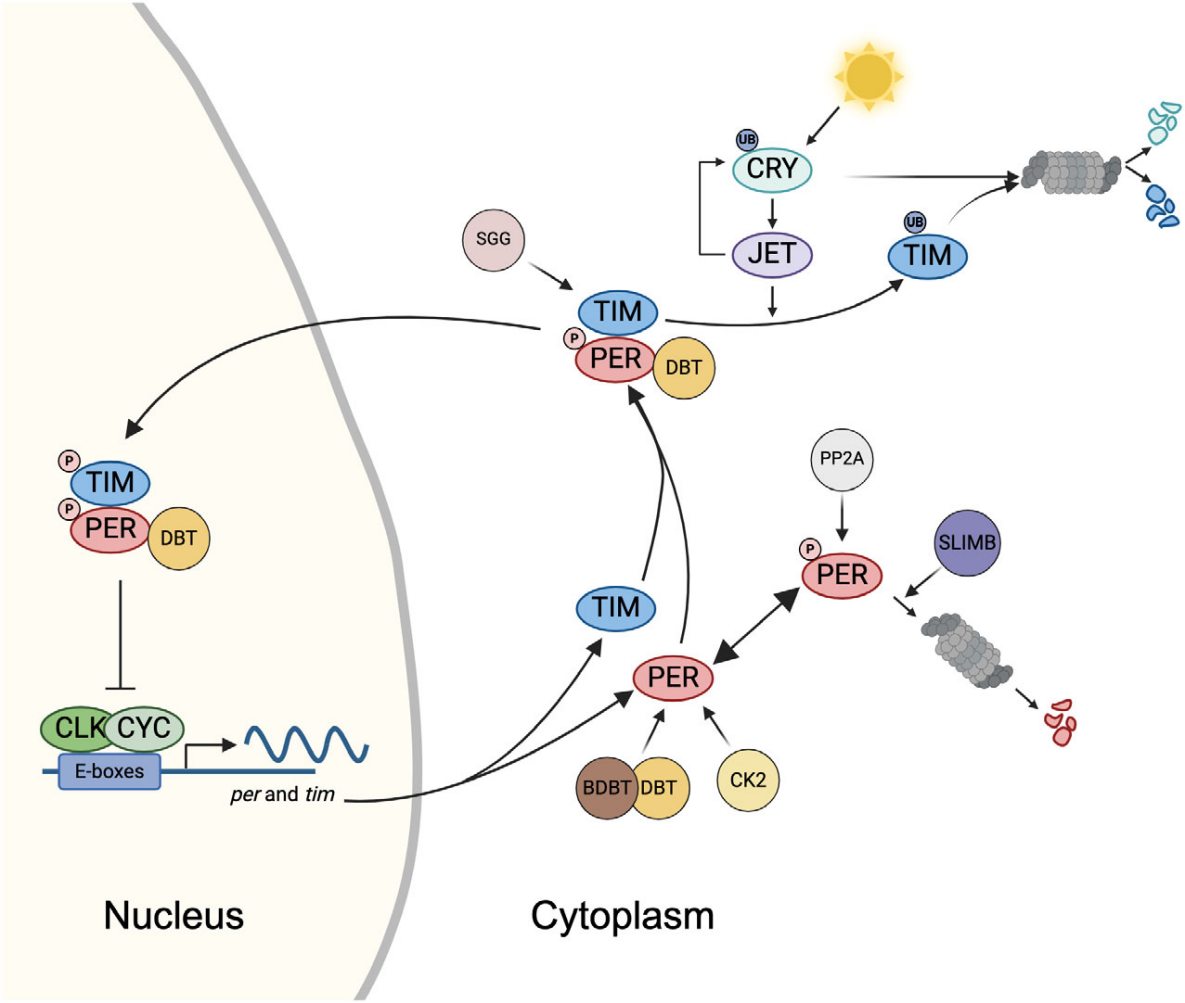
The *per/tim* feedback loop and role of CRY in molecular clock resetting. During the late day–early night, the dimer CLOCK (CLK)/CYCLE (CYC) activates the expression of *period* (*per*) and *timeless* (*tim*) genes. Post‐translationally modified PER and TIM proteins accumulate in the cytoplasm during the night and form heterodimers, stabilizing them and enabling their transport to the nucleus, where they inhibit their own transcription. In the presence of light, the circadian photoreceptor CRYPTOCHROME (CRY) binds TIM and promotes its proteasomal degradation through a mechanism that involves the F‐box protein JETLAG (JET). When exposed to light, CRY also becomes a substrate for JET, which initiates its ubiquitination and degradation via the proteasome.

A schematic representation of the *per/tim* feedback loop, with the role of CRY in molecular clock resetting, is reported in Fig. 1.

### Central and peripheral oscillators

The circadian clock consists of a central brain pacemaker, that regulates daily activity rhythms, and peripheral oscillators dispersed throughout the body, that are highly coordinated to produce daily rhythms in physiology and behavior.

The circadian circuitry in *Drosophila* includes a master clock (pacemaker) operating with a network of 150 clock neurons, which are divided into 7 groups for each cerebral hemisphere: 3 groups of dorsal neurons (DN1, DN2, and DN3) and 4 groups of lateral neurons. These neurons are further categorized into three subgroups: lateral posterior neurons (LPNs), ventral lateral neurons (LNvs), and dorsal lateral neurons (LNds). Based on their relative size, the ventral lateral neurons are classified as small and large ventral lateral neurons (s‐LNvs and l‐LNvs, respectively) [19]. Among the clock neurons in the brain, s‐LNvs and LNds play specific roles in controlling morning and evening activity, respectively. [20]. Current research indicates that the circadian circuitry is far more intricate, consisting of 17 different categories of clock neurons [21], that create synaptic networks and communicate with both non‐clock partners and each other [22, 23, 24].

Peripheral clocks are widely distributed within the fly, including visual structures, antennae, proboscis, fat body, prothoracic gland, testis or cuticle, and control rhythms in phenotypes such as visual sensitivity, electroretinogram, olfaction, gustatory physiology, feeding, eclosion, sperm release, and cuticle deposition (reviewed in ref. [25]). In contrast to higher animals, in *Drosophila* and insects in general most peripheral oscillators are autonomous and can be synchronized directly by common entraining signals such as light or temperature. In addition, although the molecular oscillation is based on TTFLs, peripheral clocks differ from the central pacemaker for several reasons. The main difference is that in most peripheral tissues, for example, in the eye and the antennae, CRY also acts as a transcriptional repressor for *per* and *tim* expression, similar to mammalian CRY proteins [26] (reviewed in ref. [27]).

In this review, we focus on the visual system in the adult fly, addressing circadian oscillations in several visual structures and emphasizing the importance of a functional circadian clock in this organ.

## Anatomy of the visual system

**Fig. 2:**
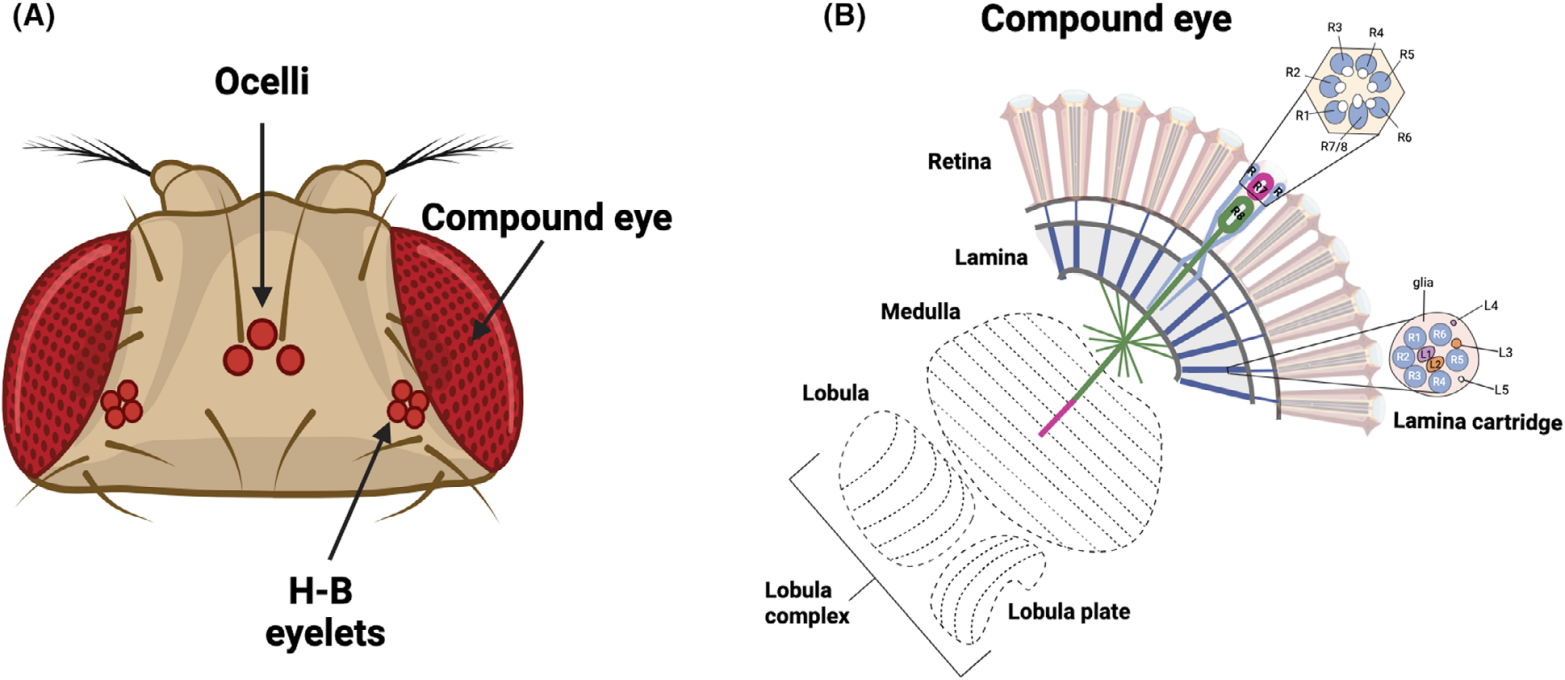
The adult *Drosophila* visual system. (A) The adult visual system of the fruit fly *Drosophila melanogaster* contains seven photoreceptive structures: two compound eyes, a pair of Hofbauer–Buchner (H‐B) eyelets, and three ocelli. (B) The compound eye is depicted in a schematic illustration, showing the retina and the four optic ganglia (lamina, medulla, lobula, and lobula plate). Within the retina, individual ommatidia accommodate photoreceptors R1–R8. In a cross‐section through the ommatidia, it is evident that the peripheral photoreceptors, R1–R6, are arranged in hexagonal patterns and cover the entire length of the retina. The inner photoreceptors, R7–R8, are situated in the center, with R7 positioned atop R8. In a cross‐section through the lamina, it can be observed that the axons of R1–R6 connect with lamina neurons to form synaptic units known as ‘cartridges’. These cartridges comprise R1–R6 input terminals and five lamina neurons (L1–L5) surrounded by glial cell processes (gl). The axons of cells R7–R8 within the inner layers extend through the lamina and end in two separate neuropil layers within the medulla.

The adult *Drosophila* visual system consists of 2 compound eyes, 3 ocelli located on the top of the fly's head, and Hofbauer–Buchner (H‐B) eyelets, 2 bilaterally symmetric clusters of photoreceptors underneath the retina (Fig. 2A). Each structure has a different phototransduction mechanism and plays a different role in the regulation of vision and rhythmic phenotypes.

## Compound eyes

Compound eyes are formed of the retina and the optic lobes divided into three neuropils—the lamina, the medulla, the lobula with the lobula plate (Fig. 2B).

### Retina

Retina photoreceptors are specialized neuronal cells regularly organized in ommatidia, cone‐structured photoreceptive units arranged in a hemispherical shell, each containing 8 types of neuronal photoreceptor cells (R1‐R8), with specific locations and photopigment expression, in addition to nonneuronal supporting cells. The six outer photoreceptors (R1‐R6), which are involved in motion detection [28], express rhodopsin 1 (Rh1) [29, 30] and terminate in the lamina, where they transfer signals to the lamina interneurons. The inner photoreceptors that terminate in the medulla and are involved in color‐related vision [31] are the R7, which expresses rhodopsin 3 or 4, and the deeper R8, which expresses Rh3, Rh5, or Rh6 [31]. Rh1 is sensitive to a broad spectrum of light, from blue–green to UV, Rh3 and Rh4 recognize UV light, and Rh5 and Rh6 recognize blue and green light, respectively [32]. Based on the rhodopsin variants expressed in the inner photoreceptors, ommatidia can be distinguished as: *yellow*, with the R7 expressing Rh4 and the R8 expressing Rh6; *pale*, in which the R7 expresses Rh3 and the R8 expresses Rh5; *dorsal rim area*, with both R7 and R8 expressing Rh3 [32]. Phototransduction is mediated by canonical G‐protein coupled signaling pathway, which requires phospholipase C (encoded by *norpA* gene) [33], and however, there are evidences that circadian synchronization via Rh5 and Rh6 requires norpA‐independent pathway [34, 35].

### Lamina

The first optic neuropil, called the lamina, has a very regular structure. It is composed of synaptic modules known as cartridges, which include R1‐R6 axon terminals, R7 and R8 axons, lamina monopolar interneurons L1‐L5 dendrites, processes of 1–2 amacrine cells, and processes of C1, C2, and T1 medullar cells. Each cartridge is surrounded by 3 epithelial glial cells. Photoreceptor R1‐6 cells form two types of synaptic connections: multiple contact tetrad synapses with L1, L2, and L3 and amacrine or glial cells and feedback synapses with lamina interneurons L2 and amacrine cells. Tetrad synapses transmit light signals from photoreceptors to deeper parts of the visual system. The function of feedback synapses is still not well‐known; however, they are excitatory and can modulate photoreceptor activity and increase photoreceptor sensitivity during dim light [36].

### Medulla

The medulla represents the largest structure in the optic lobe, with an estimated 40 000 neurons organized into 10 parallel layers (M1‐M10) orthogonal to the orientation of photoreceptor projections. The medulla can be divided into two regions: the upper M1‐M6 layers, which receive projections from R7 and R8, and the lower layers M7‐M10, which are devoid of photoreceptor projections. R7 and R8 differ in the depth of their termination and display different immunoreactivities. R7 neurons are possibly GABAergic, while R8 neurons use histamine for motion detection and acetylcholine for circadian photoentrainment [37, 38].

According to their morphologies and connections, different kinds of medullar neurons can be identified: intrinsic medulla neurons (Mi), transmedulla neurons (Tm, connecting the medulla and lobula), and Y‐shaped transmedulla neurons (TmY, connecting the medulla, lobula and lobula plate). It has been hypothesized that these neurons function as a split of visual signals into different layers specialized in color vision and motion‐detection pathways [39].

### Lobula complex

Of the last two deeper retinotopic neuropils, the *lobula* exhibits a cortex‐like organization, with ‘palisades’ formed by lobula columnar neurons (LCNs), comparable to pyramidal cells of the mammalian cortex. The exact composition and function of the lobula are still not completely understood, although it is predicted to be sensitive to object features, such as orientation, texture, and color [40].

The *lobula plate* is a tectum‐like neuropil containing large tangential neurons (LPTC), that can be grouped into horizontal (HS) and vertical (VS) systems according to the preferred direction of their dendrites. They receive uncrossed axons from the medulla and are sensitive to the orientation and direction of optic flow [41].

## Ocelli

The least known visual structures are the ocelli, which are 3 eyes located at the top of the head [42]. Every ocellus contains approximately 80 photoreceptor cells that express UV‐sensitive rhodopsin 2, cone cells, and pigment cells. Photoreceptor axons terminate directly in the optic lobes, namely, in the lobula and lobula plate. The phototransduction cascade in the ocelli is based on TRP and RdgB, but it is norpA—independent, providing an alternative pathway to the main pathway observed in retina photoreceptors. It is known that the ocelli play a role in sensing the horizon, flight stabilization, entrainment of the circadian clock, color choice, and phototaxis [43]. In general, they are involved in the detection of polarized light and small changes in the light intensity. Interestingly, this structure is typical for flying insects but not grounded, with few exceptions [44].

## Hofbauer–Buchner eyelets

Hofbauer–Buchner (H‐B) eyelets are 4 photoreceptors (in every hemisphere) located between the retina and the first optic neuropil, called the lamina. They originate from larval cholinergic visual structures called Bolwig organs, which contain two types of photoreceptors—eight expressing rhodopsin 6 (Rh6) and four expressing rhodopsin 5 (Rh5) [45]. Adult H‐B eyelets express only Rh6 [46, 47] and use both acetylcholine and histamine as neurotransmitters [42, 48, 49]. H‐B eyelets have been described as circadian photoreceptive organs [42, 48] involved in the entrainment of long and short days [43, 46]. This is possible because the terminals of H‐B eyelets reach the accessory medulla, the area where pacemaker cells are located. The H‐B can form direct synaptic connections with pigment dispersing factor (PDF)‐expressing ventral lateral neurons (LNVs) [42, 45, 46]. In the morning, eyelets activate the s‐LNvs through acetylcholine [49, 50, 51] and inhibit the l‐LNvs via histamine [51]. This communication allows synchronization of TIM and PER expression in the s‐LNv [52], l‐LNv and DN1s [53] under external light conditions.

## Daily rhythms of the visual system

### In gene expression

Retina photoreceptors express the following clock genes: *period* (*per*), *timeless* (*tim*) [54, 55] *Clock* (*Clk*) [55], *cycle* (*cyc*) [3] and *cryptochrome* (*cry*) [55]. *per* and *tim* show the maximum gene expression during the night at ZT16, while *Clk* and *cry* show the greatest gene expression during the day at ZT4. This daily pattern is maintained in constant darkness conditions (DD) with a smaller amplitude, but it is lost in *per* ^*0*^ mutants [55]. PER protein levels in photoreceptor nuclei are similar, with the greatest changes occurring at the end of the night and beginning of the day, and the protein is almost undetectable during the day [54, 56]. Peripheral oscillators in the retina seem to be independent of pacemaker cells, as mutations in *Pigment dispersing factor* (*Pdf*), the main clock neurotransmitter, or silencing of *Ion Transport Peptide* (*ITP*) do not change the rhythmic pattern of clock gene expression in the retina [55]. In addition, *disco* mutants in which the retina is unconnected to the optic lobes and in effect has no signal between the retina and the central brain, still show a strong rhythm of PER immunostaining in photoreceptor cells [54].

Several clock‐controlled genes have been identified in photoreceptors as either direct or indirect targets of the CLK‐CYC heterodimer, and among these genes, many are involved in processes such as regulation of transcriptional activity and eye development [57]. The disruption of the CLK‐CYC heterodimer by the expression of the dominant negative version of Clk (Clk^DN^) in R1‐R6 photoreceptors leads to dysregulation of transcription factors' level and/or activity and to a general decrease in chromatin accessibility [57].

Synaptic active zone in the lamina is enriched in the presynaptic BRUCHPILOT (BRP), expressed in photoreceptors in rhythmic manner. Both, gene expression and BRP protein level reach maximum twice a day, at the beginning of the day, at ZT1 and at the beginning of the night, at ZT13 [55, 58, 59]. The morning peak of expression is forced by light, while the evening one is clock‐dependent [58]. The expression levels of BRP are also regulated by light. Light exposure in the morning increases BRP expression, with a mechanism requiring additional signal from the peripheral oscillators located directly in photoreceptors [49]. Light activates cryptochrome (CRY), which changes its conformation and forms complexes with BRP. These dimers are ubiquitinated and degraded, which causes decreased amount of BRP during the day [60]. This precise mechanism of light‐dependent BRP degradation is observed only in the visual system [60].

Epithelial glia in the lamina possess their own clocks [61, 62], and however, some of the rhythms observed in the physiology of the lamina glia is regulated by pacemaker and retinal oscillators. One of the examples is alpha subunit of sodium potassium pump (Atpa), whose mRNA and protein levels are rhythmic in cartridge glia [63]. The maximum expression is observed at the beginning of the day, at ZT1 and night, at ZT13, while in constant darkness morning peak is not observed and the protein level is high during the whole night. The pattern described in DD is similar to that observed in *cry* mutants under LD12:12 conditions. Glia clock disruption does not affect rhythmicity of ATPα, but photoreceptor clock is necessary to decrease the level during the night, and PDF signaling is key to force expression in the morning [64].

The compound eyes express the transcriptional coactivator and protein phosphatase eyes absent (EYA), which shows a robust oscillation over the daily cycle [65]. EYA expression levels and temporal accumulation pattern are influenced by photoperiod and temperature, with higher but delayed peak abundances occurring under shorter photoperiods [65].

Glial cells express Ebony (N‐β‐alanyl‐biogenic amine synthetase) in a rhythmic manner, with higher expression levels during the day than at night. This enzyme is involved in the metabolism of biogenic amines, such as dopamine, octopamine, histamine, tyramine, and serotonin [66, 67]. Ebony‐expressing glia are located in close proximity to clock neuron terminals in the medulla, which indicates that they can modulate pacemaker signaling [68].

### In morphology

The number of pigment granules in the R1‐R6 photoreceptors shows circadian rhythmicity, with a maximum at the end of the day and a minimum at the end of the night; however, light exposure enhances granule formation and migration to the proximal part of terminals [69].

The number of tetrad synapses in the lamina changes daily, with greater numbers occurring at the beginning of the day, at ZT1 and at night, at ZT13 (a similar pattern is observed in housefly) [70]. This rhythm is controlled by the clock; however, the number of synapses is decreased in *cry* ^*0*^ mutants [71]. The size and shape of these synapses also change daily, which is strictly related to the presynaptic protein Bruchpilot (BRP) level and isoform composition, suggesting that BRP levels affect synaptic plasticity in lamina cartridges [71, 72]. In effect, synaptic vesicle release shows daily changes as well, with a peak occurring at night [71].

In the lamina, the analysis of cartridge cross‐sections over 24 h revealed important morphological changes. Indeed, axon sizes increased during the day, with a peak at midday, then decreased to a minimum at midnight, and the presynaptic T‐bars on the membranes surrounding the photoreceptor terminals showed a peak during the day and a trough at night [73]. Additionally, the abundance of epithelial glial cell extensions exhibited time‐dependent characteristics: while shallow capitate projections were more common during the day than they were at night, deep capitate projections were twice as common at night as they were during the day [73].

The other group of lamina cells that shows daily rhythms are monopolar L2 interneurons. They receive signals from R1‐6 photoreceptors and are transmitted to deeper parts of the brain; however, they can also send signals to photoreceptors through feedback synapses to modulate their activity. In *Drosophila*, as well as in *Musca domestica*, both L1 and L2 change their axon terminals daily [74, 75]. This rhythmicity is regulated by neurotransmitters [70], light [74], and changes in the size of epithelial glia surrounding the cartridges [76, 77]. In addition, proper balance between ionic/pH between lamina compartments is necessary to maintain rhythmic changes in L1 and L2 axon size, which is provided by daily changes in V‐ATPase expression [78]. L2 interneurons also change their dendritic tree size, with the largest area of dendrites occurring at the beginning of the day [79]. L2 axons change shape from an inverted conical shape during the day to a cylindrical shape at night [75]. Nuclei are larger during the day than during the night, while L2 cell bodies do not change in size during the day or night [80]. The number of mitochondria oscillates during the day, with the maximum in the morning [81]. Finally, L2 calcium levels show cyclical changes, indicating that their activity is also rhythmic [49].

In the visual system, peripheral oscillators are located not only in photoreceptors but also in glia. They were described in astrocyte‐like glia in the distal medulla, epithelial glia in the lamina, and optic chiasm glia [51, 58, 77]. Little is known about the detailed mechanism of the clock in these peripheral oscillators. However, they regulate rhythmic changes in the size and shape of L2 axons and dendrites [77, 79] and modulate synaptic transmission through glutamate reuptake and termination of histamine metabolism. The size of epithelial glia changes daily [72]. They form invaginations called capitate projections close to photoreceptor synapses, with a functional rhythm in number corresponding to synapse rhythmicity [69].

In the visual system, the mesencephalic astrocyte‐derived neurotrophic factor DmMANF, a secreted protein ortholog of mammalian MANF and CDNF (cerebral dopamine neurotrophic factor), is widely expressed, specifically in photoreceptor cell bodies and in the lamina (lamina cortex and synaptic neuropil) [82]. In the lamina, DmMANF is expressed in glial cells and in the cell bodies of L2 interneurons [83], and although it is not rhythmically expressed, it is necessary for the rhythmic plasticity of L2 dendritic trees [83]. Indeed, L2 cell dendritic trees in the distal lamina exhibit daily rhythms in size and shape, with a peak at the beginning of the night (ZT13), and these rhythms are abolished when DmMANF is downregulated [83].

Morphological changes in H‐B eyelets are not very precisely described; however, they show daily synaptic plasticity, as the synapses formed with LNv are active only at the beginning of the day, at ZT1 [49].

### In behavior

A functional circadian clock in photoreceptor cells is obviously important for controlling visual coding efficiency in *Drosophila* and optimizing vision under different light intensity regimes (reviewed in ref. [84]). Indeed, photoreceptor sensitivity, measured via electroretinography, is strictly time‐of‐day dependent, with the maximum in the middle of the night [85]. Additionally, the optomotor response, which refers to the tendency to turn in the same direction as a moving grating, is higher during the night, with a maximum at ZT18, and this behavior is CRY‐dependent [60, 86].

CRY in R1–R6 also contributes to measuring daylight intensity*;* in fact, *Drosophila* exhibits a significant reduction in diurnal activity with increasing daylight intensity [87]. This behavior is independent of the endogenous circadian clock itself; however, it is strictly linked to the ability of flies to measure light sensitivity, which relies on the interaction between the clock protein CRY and the phototransduction cascade, which is modulated by binding with actin [87] and Ca^++^/calmodulin [88].

**Fig. 3:**
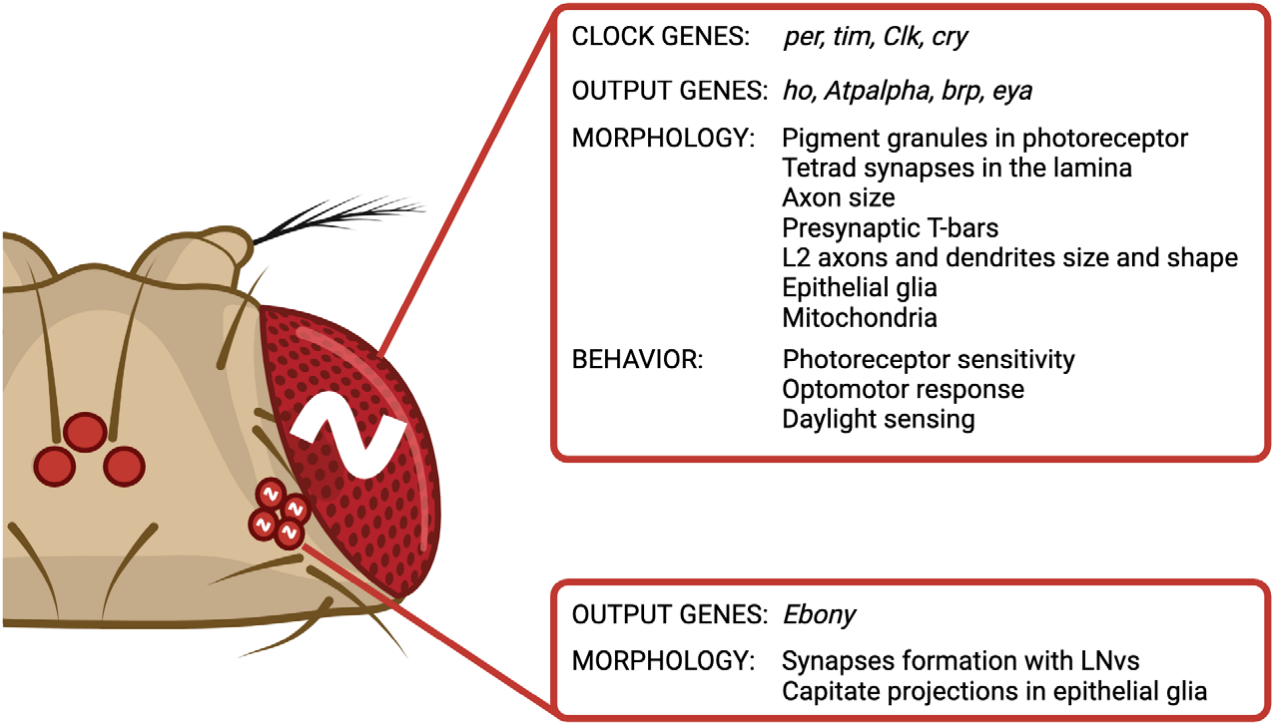
Daily rhythms of the visual system. In the compound eye, the circadian clock generates rhythms in the expression of clock and output genes, in the morphology and connections of neuronal cells and in behavior. In the HB eyelets, rhythms in output gene expression and in neuronal projections and synapses are observed. Details are provided in the main text.

A schematic of the daily rhythms of the visual system is shown in Fig. 3.

## Center *vs* periphery: The same, the similar, and the different

In the visual system, the peripheral oscillators located in photoreceptors and in glial cells show similar phase in the main clock gene and protein oscillations. *per* and *tim* mRNA levels reach their peak in the middle of the night, at ZT16 [55], a scenario in some ways distinct from pacemaker neurons, where the highest levels of *per* and *tim* gene expressions are seen earlier, at ZT13 [89]. The pattern of PER protein oscillation, that reaches the maximum at the beginning of the day, is conserved between peripheral clocks in photoreceptors and glia [54] and LNvs [54, 90]. In these clock neurons, PER oscillation is temperature‐dependent: A sharp minimum at ZT12 is observed only at 25 °C, while at lower or higher temperatures (17 or 28 °C) the levels are minimum between ZT8 and ZT16 [90]. Importantly, the rate of PER decay is faster in photoreceptor cells and glia, in which the immunofluorescence signal is completely lost between ZT8‐12, compared to clock neurons, where it is continuously detectable [54]. We speculate that this difference in PER regulation could depend on the activity of BDBT (BRIDE of DOUBLETIME), the noncanonical FK506‐binding protein that accumulates rhythmically in PERIOD and DOUBLETIME‐dependent cytosolic foci, regulating PERIOD and DBT nuclear accumulation [9]. In the eye, the focal activity of BDBT is negatively regulated by light, which acts through both CRY and the visual photoreceptor proteins [91]. Reduced BDBT foci in the eye result in increased cytosolic DBT [91], thus impacting on the kinetics of PER degradation.

A different phase between central and peripheral oscillators is observed in *Clk* expression, that in pacemaker neurons reaches maximum levels at the beginning of the day, ZT0 [89], while in the retina the maximum is observed later, at ZT4 [55]. In the retina, the oscillation of *Clk* mRNA is modulated by the circadian photoreception: indeed, in *cry* null mutants a second peak observed at ZT16, suggesting that CRY suppresses *Clk* expression at ZT16 [55].

Both photoreceptors and s‐LNvs show daily changes in synaptic plasticity. Photoreceptors show rhythmic changes in the level of presynaptic protein BRP in R1‐R6 terminals, with maximum at the beginning of the day and night [58]. This oscillation of BRP levels corresponds to the number of tetrad synapses formed between R1‐R6 photoreceptors and cells receiving the signal [71]. These rhythms are both, clock‐ and light‐dependent, in constant darkness only the evening peak is observed [58]. Similarly, s‐LNs show a very strong rhythm in the complexity of terminals in the dorsal brain, which provides different synaptic partners throughout the day [92]. However, the pattern of the oscillation differs from that of photoreceptors: A maximum is observed at ZT2 and a minimum at ZT14 [92], and the oscillation is not dependent by light, as it is maintained in constant darkness [92].

Considering that the retina is the tissue that is exposed to light the most, it is not surprising that light has a greater effect on the photoreceptor clock than it does on the central pacemaker, as demonstrated by the aforementioned examples.

The most important difference between central and peripheral clock is the role of CRYPTOCHROME [93], which is unique in the visual system, especially in the retinal cells. Firstly, co‐expression of CRY and PER in compound eyes represses CLK/CYC activity, suggesting that CRY functions as a transcriptional regulator of the expression of *per* and *tim* [26]. Second, CRY forms complexes in the visual system not just with TIM but also with proteins related to phototransduction [86], synaptic activity [60], and calcium signaling [88]. Lastly, while CRY in pacemaker cells is only active in response to light stimulation, in photoreceptors it is active during the dark and is most likely triggered by a Ca^++^/calmodulin signaling pathway [88].

## Beyond genuine rhythmicity

The presence of a circadian clock in the visual system is important not only for the generation of daily rhythms in visual behavior but also for modulating other aspects of physiology that do not exhibit proper rhythms.

The circadian clock in compound eyes plays a role in the measurement of photoperiod, as it mediates not only light but also other environmental inputs to the *Drosophila* brain. Indeed, they express EYA, a well‐known developmental factor that promotes reproductive dormancy, and ovary growth is arrested under short‐day and cold‐temperature conditions [65]. In the optic lobe, EYA colocalizes and interacts with the TIM, which mediates its stability and contributes to the seasonal physiological responses of the fly [65].

The retina is the tissue most exposed to light, and it is very vulnerable to DNA damage caused by high‐energy wavelengths and oxidative stress. One of the protective pathways is the high expression of heme oxygenase (HO), which is much greater than that in the brain. HO is an enzyme involved in heme degradation and ROS scavenging, with a cytoprotective role exerted via the control of DNA damage [94, 95]. The *ho* gene is rhythmically expressed, with the highest level occurring at the beginning of the day (ZT1) and during the night (ZT16). This pattern is maintained in constant darkness but disappears in *per* null mutants [96]. This enzyme in the retina also seems to be involved in the regulation of the molecular clock. Indeed, increased HO activity mediated by hemin feeding resulted in lower expression of *per* and higher expression of *Clk*. Opposite results, that is, increased *per* and decreased *Clk* mRNA, were observed after SnPPIX treatment (HO inhibitor) or *ho* silencing [96]. This pathway employs nitric oxide (NO), which is regulated through HO, and in effect, it affects CLOCK activity [96]. The morning peak of *ho* expression is driven by the circadian clock; however, it can also be enhanced by a light pulse in flies kept in constant darkness. Interestingly, this is possible only at specific times of day, ZT1 and CT1, but not later during the day or during the night [95]. Flies with disrupted phototransduction, *norpA* mutants, still show this regulation, that is lost in *cry^0^* mutants. To increase *ho* gene expression in the retina light pulse needs to be at least 45 min long and 120 lux intense. This phenomenon was observed for UV, blue, and very intense white light, which indicates that it is mechanism of protection against light‐dependent cell damages. Indeed, it was shown that inhibition of HO activity increases, while HO activation decreases, the amount of DNA damage [95, 97]. The number of DNA breaks after UV exposure is also time dependent, as the retina seems to be most vulnerable during the night [95]. Furthermore, the peak of *ho* expression in the middle of the night seems to be connected to the suppression of innate immunity in the visual system, as *ho* silencing in the retina increases the expression of antimicrobial peptide genes at ZT16 [98].

The circadian clock in the retina is important for the chromatin remodeling of actively expressed genes in *Drosophila* photoreceptors, as its disruption causes a general decrease in chromatin accessibility [57]. Furthermore, progressive light‐dependent retinal degeneration and oxidative stress were observed as a consequence of the downregulation of many phototransduction components whose loss leads to light‐dependent retinal degeneration. These observations clearly indicate a role for the circadian clock in the maintenance of *Drosophila* photoreceptor integrity [57].

## Conclusions

The visual system is not just a light‐sensing organ that relays light information to the brain; it also has a circadian oscillator that actively regulates retinal physiology and function.

In *Drosophila*, reciprocal interactions between the central pacemaker and the peripheral clock in the visual system have evolved. Indeed, the central clock in the brain modulates the phase and/or amplitude of the peripheral oscillator in the visual system, and on the other hand, the peripheral clock in compound eyes sends time‐related light information to central clock neurons. This synergistic interplay grants the effective synchronization of flies to both daily and seasonal changes in the environment.

Several lines of evidence support the protective role of a functional circadian clock in tissues highly exposed to DNA damage and oxidative stress, such as the retina. Through mechanisms such as chromatin remodeling, phototransduction modulation, and the control of DNA damage, the circadian clock plays a cytoprotective role that safeguards fly photoreceptor integrity.

## Conflict of interest

The authors declare no conflict of interest.

## Author contributions

MD and GMM developed the concept and wrote the manuscript.
